# Advanced Technologies for the Diagnosis of Pulmonary Tuberculosis Using Exhaled Breath Samples: A Systematic Scoping Review

**DOI:** 10.1111/tmi.70084

**Published:** 2026-01-25

**Authors:** F. L. Adão‐Araújo, B. G. Ferreira, R. Pontarolo, F. S. Tonin, S. M. Raboni

**Affiliations:** ^1^ Postgraduate Program in Internal Medicine and Health Science Federal University of Paraná Curitiba Brazil; ^2^ Postgraduate Program in Pharmaceutical Sciences Federal University of Paraná Curitiba Brazil; ^3^ Department of Pharmacy, Postgraduate Program in Pharmaceutical Sciences Federal University of Paraná Curitiba Brazil; ^4^ Pharmacy and Pharmaceutical Technology Department, Social and Legal Pharmacy Section University of Granada Granada Spain; ^5^ Department of Community Health Federal University of Paraná Curitiba Brazil

**Keywords:** electronic noses, exhaled breath samples, non‐invasive diagnosis, pulmonary tuberculosis, scoping review

## Abstract

**Objectives:**

Non‐invasive methods based on exhaled breath samples have emerged as promising alternatives for the diagnosis of pulmonary tuberculosis, especially in settings where sputum collection is challenging and laboratory infrastructure is limited. We aimed to map the evidence on advanced technologies available for detecting pulmonary tuberculosis using alveolar breath, bioaerosols, or exhaled breath condensate.

**Methods:**

A comprehensive scoping review following international guidelines was performed (OSF: 10.17605/OSF.IO/TRJ7W). Searches were conducted in PubMed, Scopus and Web of Science (June 2025). Primary studies evaluating physical or chemical diagnostic methods based on biological exhaled breath samples for detecting pulmonary tuberculosis in symptomatic patients were included. Studies' metadata, including method accuracy metrics, were extracted for diagnosis performance comparison. Evidence was synthesised using descriptive statistical and qualitative methods.

**Results:**

Overall, 19 studies published between 2010 and 2025 were included, mostly conducted in high tuberculosis‐burden countries such as South Africa (16%), China (11%) and Paraguay (11%). The most evaluated techniques were electronic nose (e‐nose) (42%), gas chromatography coupled with mass spectrometry (GC–MS/MS‐based) (21%) and machine learning applied to mass spectrometry (ML–MS) approaches (11%). Important heterogeneity in studies' design and analytical approaches was observed. All included studies used non‐invasive samples and were classified as intended for screening purposes; none aimed for confirmatory diagnosis. Among the included studies, 10 (53%) were classified as near point‐of‐care approaches. Of these, 8 (80%) met the minimum sensitivity requirement for screening tests, although only one also achieved the corresponding specificity threshold. The highest diagnostic performance reported showed a sensitivity of 98.5% (95% CI: 92.1–100) and a specificity of 100% (95% CI: 93.5–100).

**Conclusions:**

Although research on exhaled breath‐based technologies for pulmonary tuberculosis diagnosis is advancing, with some alternatives presenting moderate‐to‐high performance, their implementation still requires standardised validation, methodological improvements and field testing in real‐world settings.

## 1 Introduction

Pulmonary tuberculosis (TB), caused by *Mycobacterium tuberculosis* , remains one of the leading causes of infectious disease–related deaths worldwide, particularly in low‐ and middle‐income countries where access to timely diagnosis is limited. According to the 2024 World Health Organization (WHO) Global Tuberculosis Report, an estimated 10.8 million people developed tuberculosis in 2023 and 1.1 million died, including 161,000 deaths among people living with HIV worldwide [1]. Although treatment for TB has significantly advanced, the early identification of pulmonary cases remains a challenge, especially in underserved settings such as rural areas, regions with limited health infrastructure and among vulnerable groups, including children and people living with HIV (PLWH) [1, 2].

Traditional approaches to TB diagnosis—such as smear microscopy, culture, or nucleic acid amplification tests—are dependent on sputum samples, specialised equipment and qualified personnel, which are often unavailable in resource‐constrained settings. These infrastructural demands are frequently incompatible with the realities of high‐burden settings—such as sub‐Saharan Africa, Southeast Asia and regions of Eastern Europe and Latin America—where diagnostic services remain scarce and health systems face chronic resource constraints [3, 4]. In response, considerable research has focused on creating faster, less invasive and more accessible diagnostic alternatives to expand screening capabilities, especially in underserved populations.

Exhaled breath samples, encompassing alveolar breath, bioaerosols and exhaled breath condensate (EBC), have been investigated as viable alternatives to sputum‐based diagnostics. Alveolar breath predominantly contains gas‐phase volatile organic compounds (VOCs) originating from the deep lung and systemic circulation, reflecting host metabolic alterations and *Mycobacterium tuberculosis* –related products. Bioaerosols correspond to the particle‐rich fraction of exhaled air and may carry viable bacilli, nucleic acids, or other pathogen‐derived components, offering the potential for more direct detection of infection. In turn, EBC consists mainly of condensed water vapour and non‐volatile organic compounds (NOCs), such as metabolites, lipids and proteins, which can serve as indirect biomarkers of inflammatory and immune responses [5, 6]. Advanced technologies—including electronic noses (e‐noses), mass spectrometry and immunological methods—have been applied to these breath‐derived matrices, often in combination with machine learning (ML) algorithms to enhance discrimination between active TB and non‐TB states [7, 8].

Despite the promising potential of breathomics‐based methods, several challenges must be addressed before their widespread clinical application. Key limitations include variability in diagnostic performance and the lack of standardised breath sample collection protocols. In addition, heterogeneity in study designs and inconsistent diagnostic criteria hinder cross‐study comparability. Although previous reviews have attempted to address this topic, many are now outdated and limited in scope—focusing on specific technologies, narrow patient populations, or relying on narrative approaches [3, 9, 10]. No study has systematically mapped the landscape of breath‐based diagnostics for pulmonary TB by integrating evidence across different sample types while also evaluating point‐of‐care (POC) feasibility.

According to the 2024 WHO Target Product Profile (TPP) for tuberculosis diagnosis, POC tests are defined as diagnostic tools that do not require instruments or specific infrastructure (such as electricity, laboratory facilities, or a cold chain) and can be performed by non‐specialised staff in decentralised healthcare settings; examples include dipstick or lateral‐flow formats [2].

Thus, this study aimed to provide a comprehensive overview of breath‐based approaches for diagnosing pulmonary TB, focusing on their methodologies, diagnostic performance and real‐world applicability. It also sought to identify key gaps and challenges to support the development of effective, non‐invasive tools aligned with WHO standards and global health priorities.

## 2 Methods

This scoping systematic review was conducted according to the Joanna Briggs Institute [11] and Cochrane Collaboration's recommendations for systematic reviews of diagnostic test accuracy [12] and reported according to the PRISMA‐ScR (Preferred Reporting Items for Systematic Reviews and Meta‐Analyses extension for Scoping Reviews) [13]. The protocol is available at Open Science Framework (OSF)—DOI: 10.17605/OSF.IO/TRJ7W.

The following electronic databases were searched for references: PubMed, Scopus and Web of Science (updated in June 2025) without time or language restrictions. The full search strategy is available in Table S1. A manual search was conducted in the reference list of the included studies.

Eligibility criteria were defined according to the PCC mnemonic (Population, Concept and Context). Primary studies that evaluated physical or chemical diagnostic methods using biological exhaled breath samples for the detection of pulmonary TB in suspected symptomatic patients (any sex or age) in any clinical setting were included. Studies published in non‐Roman characters, conference abstracts, literature reviews (e.g., narrative or integrative) and in vitro or animal‐based studies were excluded. Studies focused on extrapulmonary TB were not eligible, as the review examined diagnostic methods using exhaled breath specifically for pulmonary TB.

During the initial screening, reviewers evaluated titles and abstracts of the recovered registers to identify relevant studies. These steps were performed in the Rayyan QCRI software [14].

Full‐text articles were then assessed following the eligibility criteria. The screening was conducted independently by two reviewers, with disagreements resolved by a third reviewer. Rayyan QCRI was used solely to assist in reference organization and did not influence eligibility decisions, which followed the predefined PCC criteria.

After full‐text analysis, metadata from included studies were extracted into structured Excel spreadsheets. Extracted variables included: baseline characteristics (authors, year of publication, country, study design, population, type of breath sample), diagnostic method, their results (true‐positive, TP; true negative, TN; false positive, FP; false negative, FN; sensitivity, specificity, positive predictive value—PPV, negative predictive value—NPV, area under the curve—AUC, overall accuracy, time to obtain results) and applicability.

Overall accuracy was defined as (TP + TN)/(TP + TN + FP + FN) × 100, following the information reported by each study. Time to obtain results was defined as the turnaround time from sample collection to result availability. Applicability described operational feasibility, such as portability, infrastructure needs and compatibility with POC workflows.

The reference standard used to classify participants as TB‐positive or TB‐negative was extracted for each study and categorised according to the Cochrane Collaboration's guidance for diagnostic test accuracy reviews [12]. Single, composite and multiple reference standards were defined as uniform microbiological testing, combined microbiological and clinical/radiological criteria, or differing diagnostic criteria across participant subgroups, respectively.

In addition, a structured methodological appraisal was performed based on domains recommended in the Cochrane Handbook for Diagnostic Test Accuracy Reviews, including population definition, gate design and reference standard design. The classifications, their implications for risk of bias and the methodological assessment conducted to qualify the robustness of the included studies are summarised in Table S2.

Data were extracted as reported by the authors (both in the main article and supplementary material, when available). When necessary, accuracy data from the original articles were converted and standardised based on contingency tables. For studies reporting only receiver operating characteristic—ROC curves, AUC values were manually estimated using digitised graphs via the WebPlotDigitizer software (version 4.6) [15]. This approach allowed for standardised and comparable integration of results despite the heterogeneity in data presentation across studies.

The diagnostic methods identified in the studies were categorised into broader analytical groups, with detailed descriptions of the specific techniques provided in Table 1. This categorization supported subsequent data extraction procedures. For studies with multiple phases or cohorts (e.g., calibration and validation), performance data from the validation phase were prioritised to enhance comparability and reduce the risk of overfitting associated with training or calibration stages.

**TABLE 1:** Characteristics of the included studies (*n* = 19).

Study ID	Country	Population	*N*	Sample type	Detection platform	Algorithmic model	Signal detected
Badola 2023 [16]	India	≥ 10 years old with pulmonary TB symptoms or recent exposure (mainly adults)	334	Breath	Fast IMS	AI‐based (unspecified method)	VOC fingerprint
Beccaria 2019 [17]	South Africa	≥ 18 years old with pulmonary TB symptoms; controls: symptomatic TB‐negative	50	Breath	GC–MS/MS‐based	RF, SVM, PLS‐DA	Identified VOCs
Bruins 2013 [18]	Bangladesh	> 15 years old with suspected pulmonary TB; controls: symptomatic or healthy volunteers	178	Breath	e‐nose	ANN	VOC fingerprint
Chen 2022 [19]	South Africa	Adults with suspected pulmonary TB	99	EBA	LC–MS/MS	SAM	NOC—metabolites and lipids
Fu 2023 [20]	China	18–70 years old with confirmed TB; controls: healthy or with other lung diseases	1405	Breath	ML–MS	XGBoost, SVM, RF, kNN, AdaBoost, LR, DT	VOC fingerprint
Kolk 2012 [21]	South Africa	> 18 years old with pulmonary TB symptoms	171	Breath	GC–MS/MS‐based	SVM	Identified VOCs
Mohamed 2017 [22]	Egypt	Adults with pulmonary TB; controls: healthy volunteers	623	Breath	e‐nose	ANN	VOC fingerprint
Mosquera 2022 [23]	Colombia	Children and adults with TB symptoms; controls: healthy or pneumonia	91	EBC	Immunoassay	Not applicable	NOC—lipids, proteins, LAM
Mougang 2023 [24]	Cameroon	> 18 years old with pulmonary TB; controls: healthy without respiratory symptoms	100	Breath	e‐nose	LDA	VOC fingerprint
Phillips 2010 [25]	USA, Philippines and UK	≥ 13 years old with TB symptoms; cases classified by clinical/microbiological criteria	226	Breath	GC–MS/MS‐based	LDA	VOC fingerprint
Phillips 2012 [26]	Philippines, UK and India	≥ 13 years old with TB symptoms or diagnostic findings	251	Breath	GC–MS/MS‐based	WDA	VOC fingerprint
Saktiawati 2019 [27]	Indonesia	≥ 18 years old with suspected pulmonary TB; controls: healthy volunteers	469	Breath	e‐nose	ANN	VOC fingerprint
Coronel Teixeira 2017 [28]	Paraguay	> 18 years old with suspected TB; controls: asthma/COPD and matched healthy volunteers	175	Breath	e‐nose	ANN	VOC fingerprint
Coronel Teixeira 2023 [29]	Paraguay	≥ 15 years old with pulmonary TB symptoms, referred to tertiary hospital	213	Breath	e‐nose	ANN	VOC fingerprint
Zetola 2017 [30]	Botswana	≥ 21 years old with pulmonary TB; controls: HIV‐negative asymptomatic adults	71	Breath	e‐nose	k‐NN	VOC fingerprint
Bijker 2024 [31]	Kenya	< 5 years old children with TB symptoms	79	Breath	e‐nose	ANN	VOC fingerprint
Xu 2024 [32]	China	18–70 years old with diabetes, with and without pulmonary TB	190	Breath	ML–MS	XGBoost	VOC fingerprint
Meiwes 2024 [33]	Germany and Moldova	≤ 16 years old children with pulmonary TB	10	Bioaerosol	DNA detection	Not applicable	Mtb DNA
Alfahdawi 2025 [34]	Iraq	≥ 18 years old with confirmed pulmonary TB; controls: healthy volunteers	200	Breath	Isotope‐based	Not applicable	Isotope ratio (13CO_2_)

*Note*: Studies enrolling paediatric‐only populations, adult populations, or mixed populations (children and adults) are presented together for mapping purposes only; no direct comparison of diagnostic performance across age groups was performed. Glossary of Terms and Abbreviations (related to Table 1): AdaBoost (Adaptive Boosting): Ensemble ML method that combines weak classifiers to improve performance; AI‐based: artificial intelligence–based pattern recognition, typically involving supervised or unsupervised learning; specific algorithm not reported; ANN (Artificial Neural Network): computational model inspired by the structure of the human brain, used to detect complex patterns; Breath: generic exhaled air sample obtained from spontaneous breathing, without particle enrichment or condensate separation; Bioaerosol: exhaled particle fraction (bioaerosols) collected directly from breath, typically containing airborne droplets or microbial content; DNA detection: identification of *Mycobacterium tuberculosis* genetic material in exhaled bioaerosols using DNA‐based molecular assays; DT (Decision Tree): tree‐based supervised learning algorithm used for classification tasks; EBA (Exhaled Breath Aerosol): fine particles expelled during breathing, collected for diagnostic analysis; EBC (Exhaled Breath Condensate): liquid sample obtained by condensing exhaled air, used to analyse non‐volatile compounds; e‐nose (Electronic Nose): non‐specific sensor arrays producing composite signals (‘breathprints’) analysed via pattern recognition or ML; Fast IMS (Ion Mobility Spectrometry): a rapid detection method that separates ionised molecules based on their mobility in a carrier gas; GC–MS/MS‐based: gas chromatography coupled with mass spectrometry (e.g., GC–TOF–MS, GC × GC–TOF–MS), enabling compound‐level identification of volatile organic compounds; Identified VOCs: volatile organic compounds that were chemically identified using analytical techniques such as mass spectrometry; Immunoassay: detection of immune‐related proteins or antigens in EBC using antibody‐based assays; Isotope‐based: breath test using substrates labelled with carbon isotopes to measure metabolic activity; Isotope ratio (13CO_2_): use of 13C‐urea and detection of 13CO_2_ in exhaled breath after bacterial urease activity; k‐NN (k‐Nearest Neighbours): classification algorithm that assigns a label based on the majority label of the nearest data points; LAM (Lipoarabinomannan): glycolipid from the *Mycobacterium tuberculosis* cell wall, used in immunological detection; LC–MS/MS: liquid chromatography with tandem mass spectrometry, used for profiling metabolites and lipids, especially in EBA; LDA (Linear Discriminant Analysis): technique that finds a linear combination of variables to best separate multiple classes; LR (Logistic Regression): A regression model used for binary classification tasks in ML; ML–MS: algorithms such as RF, SVM, XGBoost applied to raw or processed mass spectra (VOC fingerprint) to classify TB status; Mtb DNA: detection of *Mycobacterium tuberculosis* genetic material; NOC (non‐volatile organic compounds): non‐gaseous molecules in exhaled samples, such as metabolites, lipids, or proteins; PLS‐DA (partial least squares discriminant analysis): supervised classification technique combining PLS regression and linear discriminant analysis; RF (Random Forest): Ensemble learning method using multiple decision trees for improved classification; SAM (Significance Analysis of Microarrays): statistical method for identifying significantly different features in high‐dimensional data; SVM (Support Vector Machine): supervised learning model that finds the optimal boundary (hyperplane) between classes; VOC fingerprint: unspecified signal or pattern of volatile organic compounds detected in exhaled breath, used as a diagnostic marker without identifying individual compounds; WDA (Weighted Digital Analysis): proprietary algorithm used in some e‐nose platforms for classifying digital breath patterns; XGBoost (Extreme Gradient Boosting): highly efficient ML method based on gradient‐boosted decision trees.

Studies were also evaluated for compliance with the minimum and optimal criteria defined in the updated WHO TPPs for tuberculosis diagnostics, considering diagnostic performance, operational applicability and inclusion of priority populations [2]. The results of this assessment are summarised in Table 2. Finally, a narrative synthesis of the studies, organised by intervention and target population characteristics, was summarised in tables and figures.

**TABLE 2:** Compliance of included studies to WHO Target Product Profile (TPP) Criteria for TB screening—2024.

Study ID	Operational category	Sensitivity	Specificity	Result ≤ 2 h	HIV+ included
Badola 2023 [16]	Near‐POC	✅	❌	✅	✅
Beccaria 2019 [17]	⬜	⬜	⬜	⬜	⬜
Bruins 2013 [18]	Near‐POC	✅	❌	✅	❌
Chen 2022 [19]	⬜	⬜	⬜	⬜	⬜
Fu 2023 [20]	⬜	⬜	⬜	⬜	⬜
Kolk 2012 [21]	⬜	⬜	⬜	⬜	⬜
Mohamed 2017 [22]	Near‐POC	✅	✅	❌	❌
Mosquera 2022 [23]	⬜	⬜	⬜	⬜	⬜
Mougang 2023 [24]	Near‐POC	✅	❌	❌	✅
Phillips 2010 [25]	⬜	⬜	⬜	⬜	⬜
Phillips 2012 [26]	Near‐POC	❌	❌	✅	❌
SaktiawatiI 2019 [27]	Near‐POC	✅	❌	❌	✅
Coronel Teixeira 2017 [28]	Near‐POC	✅	❌	❌	❌
Coronel Teixeira 2023 [29]	Near‐POC	❌	❌	❌	✅
Zetola 2017 [30]	Near‐POC	✅	❌	✅	✅
Bijker 2024 [31]	Near‐POC	✅	❌	✅	❌
Xu 2024 [32]	⬜	⬜	⬜	⬜	⬜
Meiwes 2024 [33]	⬜	⬜	⬜	⬜	⬜
Alfahdawi 2025 [34]	⬜	⬜	⬜	⬜	⬜

*Note*: ✅ = Criterion met; ❌ = Criterion not met; ⬜ = Not applicable. POC: tests performed without instruments or infrastructure, usable by non‐specialised personnel. Near‐POC: instrument‐based tests that can be operated in clinics without laboratory facilities. Low‐complexity: instrument‐based tests requiring basic laboratory infrastructure at peripheral sites. Not applicable: high‐complexity laboratory instruments (e.g., mass spectrometry platforms) that cannot be used at peripheral levels. Sensitivity thresholds follow WHO TPP 2024 requirements for non‐sputum tests: ≥ 65% for POC, ≥ 75% for near‐POC and ≥ 80% for low‐complexity assays. The *specificity* threshold is > 98% for all categories.

## 3 Results

**FIGURE 1:**
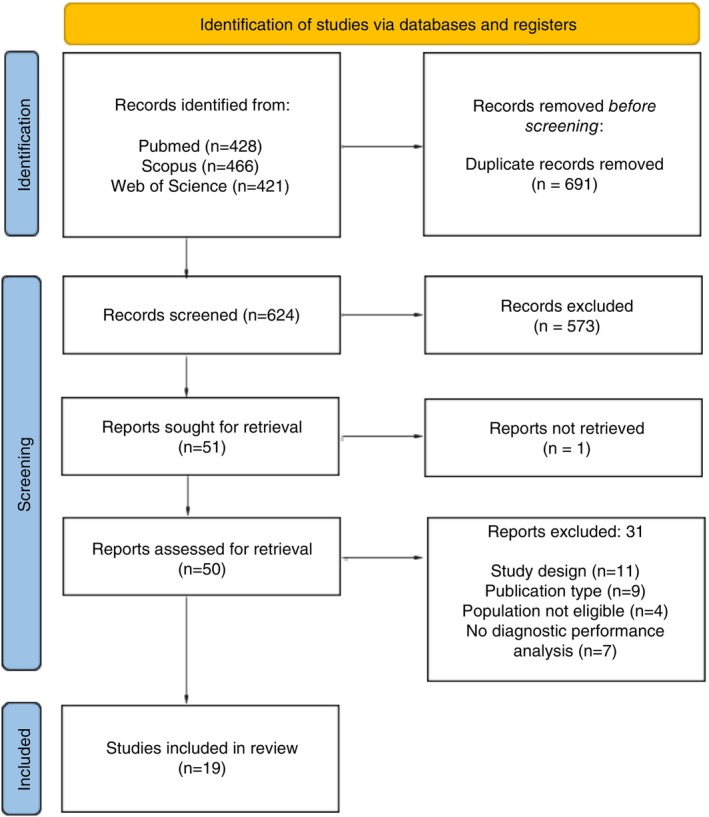
Flowchart of the study selection process, following the PRISMA‐ScR methodology. The diagram summarises the number of records identified through database searches, screened, excluded and included in the review, as well as reasons for full‐text exclusions.

The search in the databases yielded 624 records after duplicates removal, of which 573 were excluded during screening. From the remaining 51 articles, 31 studies were excluded after full‐text reading (see Table S3), leaving 19 studies for data synthesis [16, 17, 18, 19, 20, 21, 22, 23, 24, 25, 26, 27, 28, 29, 30, 31, 32, 33, 34] (Figure 1). No additional records were found by manual search.

**FIGURE 2:**
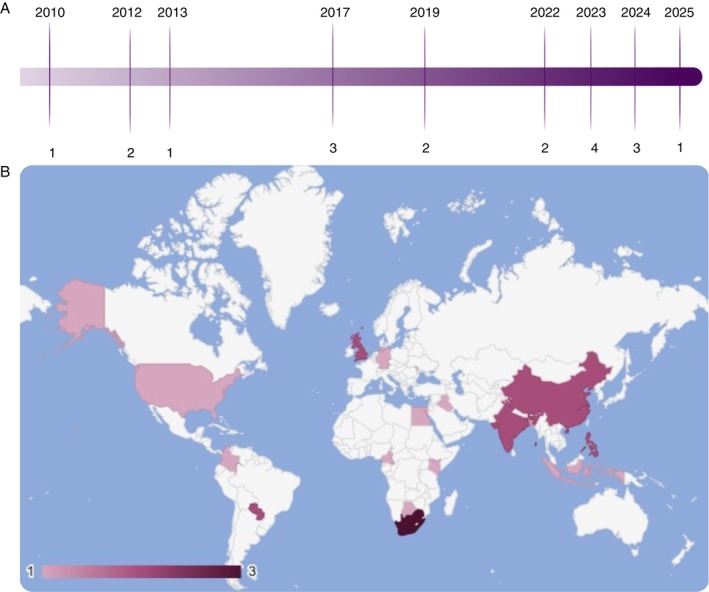
Geographic distribution and year of publication of the 19 included studies. (A) The number of studies per year (2010–2025); (B) All countries represented in the included studies, including those from multicentre investigations.

The included studies were published between 2010 and 2025. South Africa (*n* = 3; 16%) was the most frequent study setting. Five countries appeared twice—China, India, Paraguay, the Philippines and the United Kingdom (each *n* = 2; 11%). Three studies were multicentre, involving combinations of the United States, the United Kingdom, the Philippines, India, Germany and Moldova (Figure 2).

Participants were mostly adults. Only one study specifically enrolled children younger than 5 years of age (see Table 1). Comorbidities were reported in 11 out of 19 studies (58%), most commonly HIV, metabolic or cardiovascular conditions (e.g., diabetes, hypertension), pulmonary conditions (e.g., asthma, chronic obstructive pulmonary disease—COPD and pneumonia).

The studies assessed various physical and chemical methods applied to exhaled breath samples, including alveolar breath, bioaerosols and EBC. The most common platforms were e‐noses (*n* = 8; 42%), GC–MS and other MS‐based variations (*n* = 4; 21%) and ML‐MS‐based approach (*n* = 2; 11%). Five analytical methods appeared in single studies each (*n* = 1; 5%): DNA detection, immunoassay, ion mobility spectrometry (IMS), isotope‐based breath test and LC–MS/MS.

In terms of computational approaches, ML techniques were predominant (16/19; 84%). Traditional ML algorithms accounted for 8 studies (42%), while artificial neural networks (ANN) were applied in 6 studies (32%). Among the specific algorithms, Support Vector Machines (SVM) (3; 16%), Random Forest (2; 11%) and k‐Nearest Neighbours (kNN) (2; 11%) were the most frequently used. One study employed a hybrid model combining multiple algorithms, and another applied an unspecified artificial intelligence (AI)‐based pattern recognition approach (Table 1).

The methodological quality appraisal revealed substantial variability across studies. Only a minority of studies justified sample size or reported pre‐registered statistical analysis plans. In several studies, true‐negative participants originated from populations different from those of true‐positive cases, reflecting two‐gate or multi‐group designs and limiting generalizability. These findings, based on Cochrane Handbook domains, are summarised in Table S2.

As this scoping review aims to map the evidence, diagnostic performance metrics are reported descriptively at the study level and are not intended for cross‐population comparison.

**FIGURE 3:**
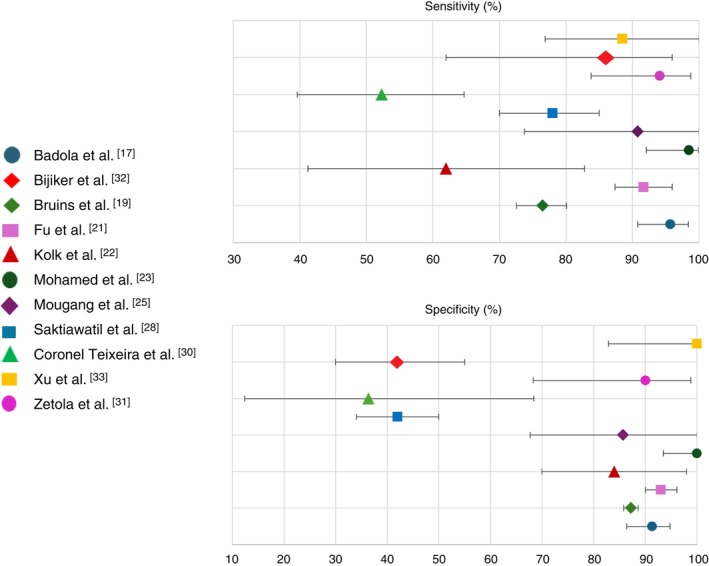
Sensitivity (left) and specificity (right) with 95% confidence intervals for diagnostic accuracy of exhaled breath‐based tests across included studies. Each point represents a single study, and error bars reflect the reported or extracted 95% confidence intervals. Colour and shape coding identify individual studies as indicated in the legend below the plots.

Among the 17 (89%) studies reporting both sensitivity and specificity, 11 (58%) provided complete data, including 95% confidence intervals. Figure 3 shows the variation in sensitivity (52.3% to 98.5%) and specificity (36.4% to 100%) reported in the studies. A full summary of reported values, including sensitivity, specificity and 95% confidence intervals, is provided in Table S4. Sensitivity and specificity varied widely across studies and analytical platforms, and a complementary descriptive visualisation of these values is presented in Figure S1. The highest diagnostic performances were reported in studies using e‐nose technologies [24, 25, 30], ion mobility spectrometry [16] and ML applied to mass spectrometry data [23].

PPV ranged from 28.9% [32] to 96.0% [30], while NPV ranged from 69.9% [28] to 98.2% [24]. High PPVs were observed in technologies with high specificity, particularly in populations with intermediate to high pulmonary TB prevalence, as reported by Badola et al., Fu et al. (XGBoost model), Zetola et al. and Coronel Teixeira et al. [16, 20, 28, 30]. Conversely, low PPVs were consistently found in studies with limited specificity, as in Saktiawati et al. and Phillips et al. [25, 27]. The highest NPVs were observed in studies reporting high sensitivity and a low proportion of false negatives, including Badola et al., Fu et al. and Mougang et al. [16, 20, 24].

Diagnostic accuracy varied from 44.6% [29] to 99.2% [22]. The best results were achieved by technologies based on electronic sensors and immunochemical methods, with Mohamed et al. (e‐nose) and Mosquera‐Restrepo et al. (immunoassay) reporting accuracy levels above 96.0% [22, 23]. AUC values spanned from 0.40 [29] to 1.00, with several approaches reporting very high AUCs (≥ 0.96), including Beccaria et al. (Random Forest), Fu et al. (Random Forest and XGBoost models) and Mosquera‐Restrepo et al. (immunoassay) [17, 20, 23]. In general, Random Forest and XGBoost models were associated with the strongest discriminatory performances. Studies reporting perfect accuracy or AUC under idealised conditions were included in the review and their results were reported; however, these findings were not interpreted as indicative of real‐world diagnostic performance due to limitations in external validity.

Based on their reported diagnostic performance relative to the WHO TPP thresholds, all 19 included studies were interpreted as having pulmonary TB screening potential [16, 17, 18, 19, 20, 21, 22, 23, 24, 25, 26, 27, 28, 29, 30, 31, 32, 33, 34]. None of the studies demonstrated performance consistent with use as a confirmatory diagnostic test (TPP Diagnostic).

Regarding specimen type, all studies used non‐invasive respiratory samples, including alveolar breath [16, 17, 18, 20, 21, 22, 24, 25, 26, 27, 28, 29, 30, 31, 32, 34], EBC [23], which is consistent with one of the operational criteria defined in the WHO TPP for screening tests. Other operational requirements specified in the TPP (e.g., time to result, instrumentation, power source and operating conditions) were not consistently reported across studies. A detailed summary of diagnostic performance and operational classification is provided in Table 2.

Only one near‐POC study—Mohamed et al. [22]—fulfilled both minimum sensitivity and specificity requirements within its operational category. However, it did not meet the time‐to‐result criterion (≤ 2 h) and did not include PLWH. Although several near‐POC studies, particularly those based on electronic nose technologies, reported sensitivity compatible with screening use, none met the corresponding specificity threshold. Similarly, few studies reported turnaround times ≤ 2 h, and none simultaneously fulfilled minimum sensitivity and specificity criteria. Among studies including PLWH, none met both minimum performance requirements according to the WHO TPP [16, 18, 24, 26, 27, 28, 29, 30, 31].

## 4 Discussion

This scoping review synthesised findings from 19 primary studies investigating advanced, non‐invasive technologies for the diagnosis of pulmonary TB using exhaled breath samples. While previous reviews have addressed aspects of this field [3, 9, 10], they were often constrained by narrow populations, technological scope, or narrative synthesis. In contrast, the present review systematically maps the landscape of breath‐based diagnostics across different exhaled breath sample types and analytical platforms, while also considering POC feasibility and alignment with 2024 WHO's TPP. A key strength of this review is the systematic and transparent assessment of methodological quality alongside reported diagnostic performance. By explicitly evaluating critical domains such as reference standards, participant comparability and sample size justification, the review provides a balanced and cautious interpretation of exhaled‐breath–based diagnostic approaches for pulmonary tuberculosis. Moreover, the focused inclusion of breath‐based technologies applied exclusively to pulmonary TB enhances the clinical relevance of the findings and offers practical guidance for the design of future diagnostic accuracy studies.

The methodological quality of the included studies varied substantially, with several reports lacking sample size justification, using heterogeneous reference standards, or recruiting true‐positive and true‐negative participants from different populations, which may limit the generalizability of their reported diagnostic performance.

Although some studies enrolled paediatric populations, the available evidence is heterogeneous and predominantly derived from adult cohorts; therefore, age‐stratified performance comparisons were outside the scope of this scoping review and remain an important gap for future research.

Methodological heterogeneity remains a critical barrier to the reproducibility and clinical translation of breath‐based diagnostics. Differences in sample collection protocols, sensor technologies, environmental control and data analysis pipelines contribute to inconsistent performance across studies. As highlighted by Rodríguez‐Pérez et al., diagnostic models often suffer a substantial drop in classification accuracy when applied to independent cohorts—falling from 85% to as low as 41%—reflecting poor generalizability and potential overfitting [35]. In TB‐specific applications, Coronel Teixeira et al. reported only 50% accuracy when externally validating an e‐nose platform, underscoring the limitations of models developed under optimised research conditions [29]. Furthermore, Westphal et al. emphasised that pre‐analytical factors such as recent diet, humidity and sampling procedures can significantly alter the VOC profile in exhaled breath [36]. This is consistent with findings by Kirwan et al., who advocate for standardised biobanking and pre‐analytical processing protocols to ensure reproducibility across metabolomics studies [37].

Despite these methodological limitations, a subset of technologies demonstrated encouraging diagnostic performance under research conditions. When diagnostic accuracy was assessed according to the WHO TPP for non‐sputum‐based tests, only one near‐POC study met both minimum performance thresholds. Mohamed et al. [22] reported a sensitivity of 98.5% (95% CI: 92.1–100) and specificity of 100% (95% CI: 93.5–100), fulfilling the near‐POC sensitivity (≥ 75%) and specificity (> 98%) requirements. However, this study did not meet the TPP time‐to‐result criterion (≤ 2 h) and did not include people living with HIV. Breath‐based approaches nonetheless offer the significant advantage of non‐invasive sample collection, making them especially promising for individuals unable to expectorate sputum, such as children or PLWH [4].

Although a subset of studies met the WHO TPP minimum performance requirements, defined by category‐specific sensitivity thresholds and a specificity > 98%—their real‐world applicability remains limited. Most studies were conducted in laboratory or tertiary care environments, lacking validation in routine screening contexts or peripheral healthcare settings [16, 17, 18, 19, 20, 21, 22, 23, 24, 25, 26, 27, 28, 29, 30, 31, 32, 33, 34]. This methodological setup likely contributed to performance overestimation, as optimised conditions and selective participant inclusion increase the risk of spectrum bias and limit generalizability to clinical populations [37].

From an operational perspective, most breath‐based diagnostic technologies were classified as near‐POC or low‐complexity assays. In several studies, operational barriers were reported, including limited device portability, dependence on a stable power supply and semi‐automated workflows. These limitations echo broader challenges described in the breath diagnostics literature, particularly regarding the feasibility of deploying such technologies in low‐resource settings [5].

Machine learning was applied in most studies (84%), yet only a minority conducted external validation using independent datasets [21, 24, 29, 32]. Internal validation methods, such as cross‐validation, were used in nine studies [17, 18, 20, 22, 23, 27, 28, 30, 31], while six studies did not report any validation at all [16, 19, 25, 26, 33, 34]. Although internal validation is useful in early development stages, the absence of independent testing increases the risk of overfitting and limits generalizability. Then, more robust validation strategies are needed to support clinical translation [38].

Moreover, the limited inclusion of high‐risk or underserved groups restricts the external validity of findings. Only six studies included PLWH, a key WHO priority population [16, 17, 24, 27, 29, 30]. Similarly, a few investigations reported subgroup analyses by comorbidity, such as diabetes or chronic lung disease, despite the known impact of host factors on VOC profiles [39]. Paediatric populations were notably underrepresented, with only two studies focusing exclusively on children, reflecting an urgent need for age‐specific diagnostic validation [31, 33].

An additional underexplored dimension is the influence of pre‐analytical variables on breath sample composition. Diet, hydration and environmental exposures are known to alter VOC profiles substantially, yet most studies lacked standardised pre‐test protocols [40]. Six studies imposed restrictions on food intake or smoking before testing [20, 24, 28, 29, 30, 32]. Evidence from metabolomics and breathomics literature indicates that these factors can lead to high intra‐ and inter‐participant variability, thereby reducing diagnostic reproducibility across settings and populations [37, 41].

Technological heterogeneity remains a significant barrier to translating breath‐based TB diagnostics into practice. As highlighted by Wilson [41], studies on e‐nose technologies vary substantially in sensor configurations, calibration methods and ML workflows, which limit comparability and hinder standardisation across research settings.

In contrast, sputum‐based diagnostics such as Truenat and LAMP underwent structured, multi‐country clinical validation prior to WHO endorsement, following a phased evaluation strategy that supports generalizability and implementation in real‐world settings [42]. However, most breath‐based platforms—including widely used systems such as the PEN3 e‐nose and IMS devices—remain confined to single‐site studies conducted under controlled experimental conditions. This gap highlights the need for harmonised validation frameworks and multicentre studies, as previously advocated in the metabolomics field to improve the reproducibility and scalability of biomarker‐based diagnostics [37].

This review focused exclusively on diagnostic methods based on biological exhaled air samples. Therefore, approaches based on cough‐sound analysis were not eligible under the predefined Concept, whereas face‐mask systems used to capture exhaled air or bioaerosols were included when aligned with this definition.

Despite the current limitations, the non‐invasive nature of breath‐based testing offers important operational advantages. Sputum collection remains challenging in 30%–50% of individuals with suspected TB—particularly among children, older adults and PLWH—which hampers timely diagnosis in high‐burden settings [4]. In contrast, exhaled breath sampling is painless, straightforward and does not generate biohazardous waste. Additionally, integration with mobile sensors and AI algorithms presents promising opportunities for decentralised screening strategies, including potential applications in remote or home‐based contexts [35].

This review has several limitations. First, most included studies did not provide sufficient data to calculate likelihood ratios (LR+ and LR–), restricting the analysis to sensitivity and specificity. In addition, substantial variability in methodological rigour—including differences in analytical approaches, reference standards, study populations and completeness of reporting—limited comparability across studies and may have contributed to performance overestimation. Moreover, AUC values were extracted as reported by each study, without harmonisation of ROC thresholds; therefore, comparisons of AUC across studies should be interpreted with caution. Additionally, extrapulmonary TB was not included because the predefined eligibility criteria restricted the scope to technologies developed for pulmonary TB diagnosis. Finally, restriction and database indexing may have led to the underrepresentation of studies from high‐burden regions. Despite these challenges, this scoping review adhered to established evidence synthesis guidelines and offers a structured, comparative appraisal of current diagnostic technologies. It provides a foundation to inform future research, standardisation efforts and innovation pathways in breath‐based TB diagnostics.

## 5 Conclusion

Breath‐based diagnostic technologies offer significant potential as non‐invasive tools to expand access to TB screening, particularly in settings where conventional diagnostics are inaccessible or impractical. Although the current evidence base reflects a steady increase in published studies and emerging diagnostic platforms, most remain in early developmental stages. Advancing the field will require standardised protocols, external validation in diverse populations and inclusion of vulnerable groups. Future research should prioritise not only diagnostic accuracy but also feasibility, reproducibility and integration into POC workflows. Coordinated investments, interdisciplinary collaboration and alignment with global health strategies are essential to translate breath‐based diagnostics into scalable solutions for TB control.

## Funding

The authors have nothing to report.

## Conflicts of Interest

The authors declare no conflicts of interest.

## Supporting information


**Figure S1:** Overview of sensitivity (A) and specificity (B) values reported across included studies, presented for descriptive comparison.

**Data S1:** tmi70084‐sup‐0002‐supinfo.docx.

## Data Availability

The datasets generated and analysed during the current study are available from the corresponding author upon reasonable request.
