# Supplementary figures and images for: Advanced Technologies for the Diagnosis of Pulmonary Tuberculosis Using Exhaled Breath Samples: A Systematic Scoping Review
Source: Trop Med Int Health. 2026 Jan 25;31(4):397–408. doi: 10.1111/tmi.70084 (PMC13050613; doi:10.1111/tmi.70084)

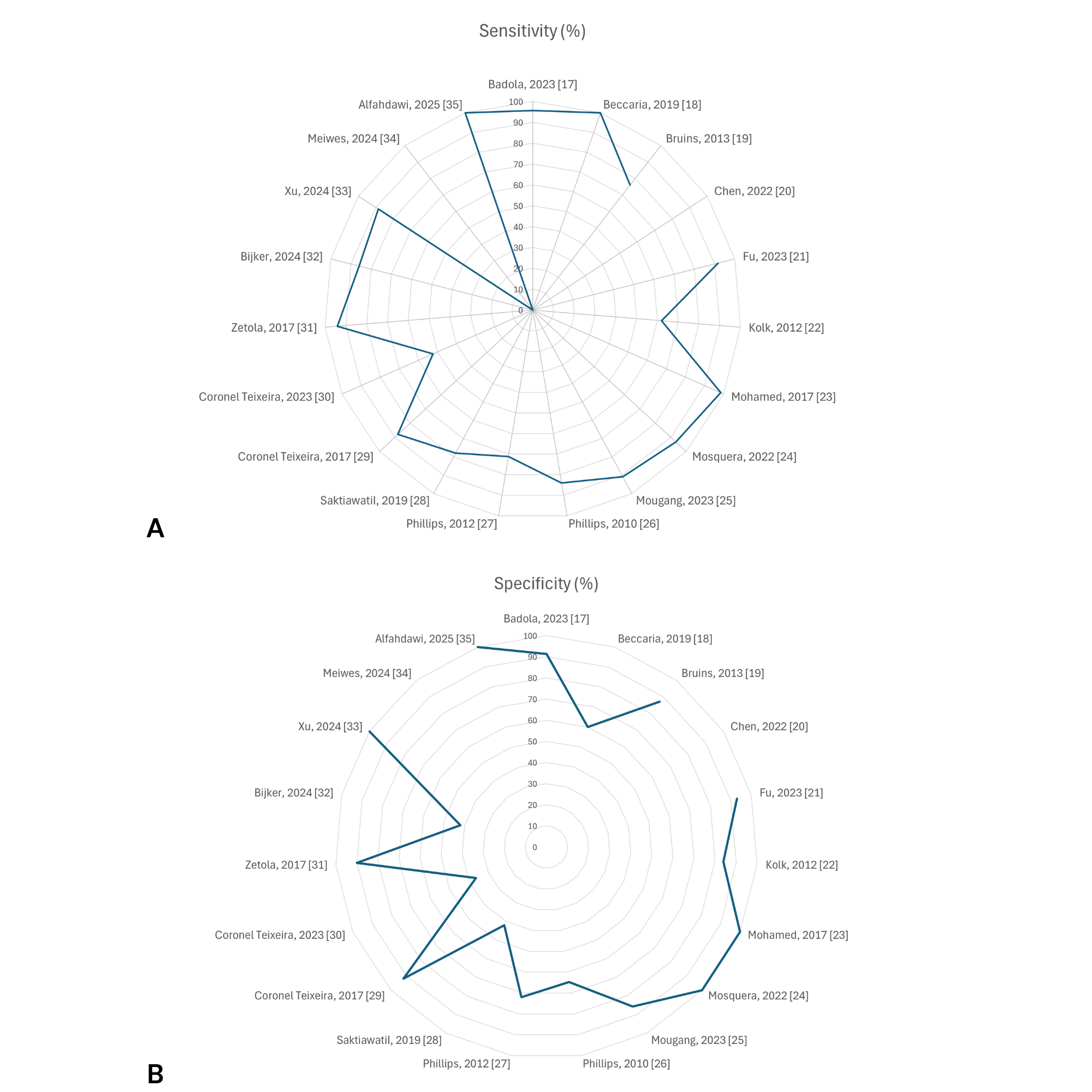

Supplement: Supplementary file 1 — Figure S1: Overview of sensitivity (A) and specificity (B) values reported across included studies, presented for descriptive comparison. [file TMI-31-397-s001.tiff]
