# Supplementary material for: Advanced Technologies for the Diagnosis of Pulmonary Tuberculosis Using Exhaled Breath Samples: A Systematic Scoping Review
Source: Trop Med Int Health. 2026 Jan 25;31(4):397–408. doi: 10.1111/tmi.70084 (PMC13050613; doi:10.1111/tmi.70084)
Supplement: Supplementary file 2 — Data S1: tmi70084‐sup‐0002‐supinfo.docx. [file TMI-31-397-s002.docx]

**Supplementary Material**

**Table S1.** Search Strategy, Databases, and Record Retrieval Summary*

| Database | Date of search | Number of records retrieved | Search query | Final query | Export method |
| --- | --- | --- | --- | --- | --- |
| PubMed (via NCBI) | June 11, 2025 | 428 | #1 Tuberculosis[MH] OR tuberculosis[TIAB] OR "Koch Disease"[TIAB]  AND  #2 Diagnosis[MH] OR diagnosis[TIAB] OR diagnostic[TIAB]  AND  #3 "Breath Tests"[MH] OR "exhaled air"[TIAB] OR exhalation[TIAB] OR breath[TIAB] | #1 AND #2 AND #3 | Send to > Citation manager > Selection: All results > Create file |
| Scopus (via Elsevier) | June 11, 2025 | 466 | TITLE-ABS-KEY (tuberculosis OR "Koch Disease”) AND  TITLE-ABS (diagnosis OR diagnostic) AND  TITLE-ABS ( "exhaled air" OR exhalation OR breath ) | : TITLE-ABS-KEY ( tuberculosis OR "Koch Disease" ) AND TITLE-ABS ( diagnosis OR diagnostic ) AND TITLE-ABS ( "exhaled air" OR exhalation OR breath ) | Export > RIS > Citation information (all boxes) and Abstract & keywords (all boxes) |
| Web of Science (via Clarivate Analytics) | June 11, 2025 | 421 | ((TS=(tuberculosis OR "Koch Disease")) AND  TS=(diagnosis OR diagnostic)) AND  TS=("exhaled air" OR exhalation OR breath) | ((TS=(tuberculosis OR “Koch Disease”)) AND TS=(diagnosis OR diagnostic)) AND TS=(“exhaled air” OR exhalation OR breath) | Export > RIS > Author, Title, Source, Abstract |

* Searches were conducted on June 11, 2025, in PubMed (n = 428), Scopus (n = 466), and Web of Science (n = 421), totaling 1,315 records. After removing 691 duplicates, 624 unique articles were screened by title and abstract. Fifty-one full-text articles were assessed for eligibility, and 19 studies were included in the final analysis. No language or date restrictions were applied.

Table S2. Methodological quality assessment included diagnostic accuracy studies based on Cochrane Handbook domains (population definition, gate design, and reference standard design).

| **Study** | **Population Definition** | **Gate Design** | **Reference Standard Design** | **Cochrane-Based Rating** |
| --- | --- | --- | --- | --- |
| Badola, 2023 [17] | Individuals ≥10 years old with TB symptoms or recent exposure; sample predominantly adult. (1 group) | ✅ Single-gate (cross-sectional cohort) | ✅ Single reference standard | ✅ Low risk of bias – Suitable |
| Beccaria, 2019 [18] | Adults ≥18 years old with symptoms suggestive of pulmonary TB; TB cases confirmed by GeneXpert MTB/RIF (culture for HIV+ cases) and symptomatic TB-negative controls. (2 groups) | ❌ Two-gate (case–control study) | ❌ Multiple reference standards | ❌ High risk of bias – Not suitable |
| Bruins, 2013 [19] | Adults ≥15 years old with suspected pulmonary TB; TB cases confirmed by sputum culture; controls were symptomatic TB-negative individuals or healthy volunteers. (3 groups) | ⚠️ Three-group (diagnostic accuracy design) | ✅ Single reference standard | ⚠️ Acceptable with limitations – Spectrum bias risk |
| Chen, 2022 [20] | Adults enrolled in a clinical trial with suspected pulmonary TB; TB status confirmed by GeneXpert MTB/RIF. (1 group) | ✅ Single-gate (cross-sectional cohort) | ✅ Single reference standard | ✅ Low risk of bias – Suitable |
| Fu, 2023 [21] | Adults 18–70 years old with confirmed pulmonary TB by Xpert/culture; controls included healthy individuals and patients with other pulmonary diseases. (3 groups) | ⚠️ Three-group (diagnostic accuracy design) | ⚠️ Composite reference standard | ⚠️ Acceptable with limitations – Spectrum bias risk |
| Kolk, 2012 [22] | Adults >18 years old with suspected pulmonary TB based on symptoms; all participants tested by culture and classified as TB or non-TB. (1 group) | ✅ Single-gate (cross-sectional cohort) | ✅ Single reference standard | ✅ Low risk of bias – Suitable |
| Mohamed, 2017 [23] | Adults with newly diagnosed pulmonary TB confirmed by smear microscopy; controls were healthy asymptomatic volunteers. (2 groups) | ❌ Two-gate (case–control study) | ⚠️ Composite reference standard | ❌ High risk of bias – Not suitable |
| Mosquera, 2022 [24] | Adults and children with pulmonary symptoms suggestive of TB, including confirmed and probable cases; controls included healthy adults, healthy children, and adults with bacterial pneumonia. (3 groups) | ⚠️ Three-group (diagnostic accuracy design) | ⚠️ Composite reference standard | ⚠️ Acceptable with limitations – Spectrum bias risk |
| Mougang, 2023 [25] | Adults ≥18 years old; TB confirmed by TB-LAMP; controls were healthy individuals without TB symptoms or respiratory disease. (2 groups) | ❌ Two-gate (case–control study) | ⚠️ Composite reference standard | ❌ High risk of bias – Not suitable |
| Phillips, 2010 [26] | Symptomatic individuals ≥13 years old at high risk of TB recruited in clinical settings; all classified as TB or non-TB using microbiological, radiological, and clinical criteria. (1 group) | ✅ Single-gate (cross-sectional cohort) | ⚠️ Composite reference standard | ⚠️ Acceptable with limitations – Spectrum bias risk |
| Phillips, 2012 [27] | Individuals ≥13 years old with symptoms or diagnostic findings suggestive of TB; cases and controls recruited separately. (2 groups) | ❌ Two-gate (case–control study) | ⚠️ Composite reference standard | ❌ High risk of bias – Not suitable |
| SaktiawatiI, 2019 [28] | Adults ≥18 years old with suspected pulmonary TB recruited from clinics and hospitals; controls were healthy volunteers without TB symptoms. (2 groups) | ❌ Two-gate (case–control study) | ⚠️ Composite reference standard | ❌ High risk of bias – Not suitable |
| Coronel Teixeira, 2017 [29] | Adults ≥18 years old with suspected pulmonary TB; controls included asthma/COPD patients and socioeconomically matched healthy volunteers. (3 groups) | ⚠️ Three-group (diagnostic accuracy design) | ⚠️ Composite reference standard | ⚠️ Acceptable with limitations – Spectrum bias risk |
| Coronel Teixeira, 2023 [30] | Adults ≥15 years old with symptoms suggestive of TB referred to a tertiary hospital; all participants classified by culture, GeneXpert, smear, or clinical diagnosis. (1 group) | ✅ Single-gate (cross-sectional cohort) | ⚠️ Composite reference standard | ⚠️ Acceptable with limitations – Spectrum bias risk |
| Zetola, 2017 [31] | Adults ≥21 years old with microbiologically confirmed pulmonary TB; controls were healthy HIV-negative adults without respiratory symptoms. (2 groups) | ❌ Two-gate (case–control study) | ⚠️ Composite reference standard | ❌ High risk of bias – Not suitable |
| Bijker, 2024 [32] | Children <5 years old with symptoms suggestive of pulmonary TB. (1 group) | ✅ Single-gate (cross-sectional cohort) | ⚠️ Composite reference standard | ⚠️ Acceptable with limitations – Spectrum bias risk |
| Xu, 2024 [33] | Adults 18–70 years old with diabetes mellitus, with and without pulmonary TB. (2 groups) | ❌ Two-gate (case–control study) | ❌ Multiple reference standards | ❌ High risk of bias – Not suitable |
| Meiwes, 2024 [34] | Children ≤16 years old with clinically or bacteriologically confirmed pulmonary TB. (1 group) | ✅ Single-gate (cross-sectional cohort) | ⚠️ Composite reference standard | ⚠️ Acceptable with limitations – Spectrum bias risk |
| Alfahdawi, 2025 [35] | Adults ≥18 years old with confirmed pulmonary TB; controls were healthy volunteers. (2 groups) | ❌ Two-gate (case–control study) | ⚠️ Composite reference standard | ❌ High risk of bias – Not suitable |

**Legend:**

✅ = Low risk / appropriate design

⚠️ = Acceptable with methodological limitations

❌ = High risk of bias

**Population definition** describes how participants were recruited and classified, including whether TB-positive and TB-negative individuals originated from the same clinically relevant population. **Gate design** follows Cochrane terminology: single-gate (1 group) indicates a single cohort of individuals with suspected pulmonary TB in which TB-positive and TB-negative participants are identified using the same eligibility criteria; two-gate (2 groups) refers to case–control designs with separately recruited TB cases and non-TB controls; three-group designs include TB cases and two distinct non-TB comparator groups.

**Reference standard design** was classified as single when all participants were evaluated using the same microbiological reference standard, and as multiple when different reference standards were applied across participant subgroups. The final **Cochrane-based rating** represents a qualitative synthesis of these domains and does not imply study exclusion.

**Table S3.** Excluded Studies After Full-Text Review (n=31)*

| **Study** | **Year** | **Title** | **Reason for exclusion** |
| --- | --- | --- | --- |
| Schreiber et al. | 2002 | Mycobacterium tuberculosis gene-amplification in breath condensate of patients with lung tuberculosis | No diagnostic performance analysis (molecular detection only) |
| Adams et al. | 2008 | Reagentless detection of Mycobacteria tuberculosis H37Ra in respiratory effluents in minutes | Study design (non-clinical experiment) |
| McNerney et al. | 2010 | Field test of a novel detection device for Mycobacterium tuberculosis antigen in cough | No diagnostic performance analysis (pilot study) |
| Fens et al. | 2013 | Diagnosis Of Active And Smear-Negative Tuberculosis By Exhaled Breath Analysis | Publication type |
| Nakhleh et al. | 2013 | Artificial olfactory system based on nanomaterials-based sensors for early detection of tuberculosis via breath test | Publication type |
| Scott-Thomas et al. | 2013 | Validating a breath collection and analysis system for the new tuberculosis breath test | Study design (non-clinical experiment) |
| Timmins et al. | 2013 | Towards Rapid Stable Isotope Breath Test Diagnosis Of Tuberculosis | Publication type |
| Nakhleh et al. | 2014 | Detecting active pulmonary tuberculosis with a breath test using nanomaterial-based sensors | Study design (controls not screened for latent TB) |
| Coronel et al. | 2015 | Diagnosis of pulmonary and extra-pulmonary tuberculosis in Paraguay using the electronic nose (ParaNose study) | Publication type |
| Sahota et al. | 2016 | A simple breath test for tuberculosis using ion mobility: A pilot study | Population not eligible (included extrapulmonary TB) |
| Mosquera-Restrepo et al. | 2017 | Fatty acid derivative, chemokine, and cytokine profiles in exhaled breath condensates can differentiate adult and children paucibacillary tuberculosis patients | No diagnostic performance analysis (biomarker detection only) |
| Beccaria et al. | 2018 | Preliminary investigation of human exhaled breath for tuberculosis diagnosis by multidimensional gas chromatography - Time of flight mass spectrometry and machine learning | No diagnostic performance analysis (proof-of-concept) |
| Morozov et al. | 2018 | Non-invasive approach to diagnosis of pulmonary tuberculosis using microdroplets collected from exhaled air | No diagnostic performance analysis (proof-of-concept) |
| Valsalan et al. | 2018 | Iot based breath sensor for mycobacterium tuberculosis | Study design (non-clinical experiment) |
| Verma et al. | 2018 | A Pilot Study Exploring The Utility Of Breath Analysis In The Diagnosis Of Adult Pulmonary Tuberculosis | Publication type |
| Zheng et al. | 2018 | Bacterial pathogens were detected from human exhaled breath using a novel protocol | Population not eligible (mixed respiratory infections; not TB diagnostic study) |
| Berna et al. | 2019 | Breath Collection from Children for Disease Biomarker Discovery | Study design (protocol) |
| Sovershaeva et al. | 2019 | History of tuberculosis is associated with lower exhaled nitric oxide levels in HIV-infected children | Study design (physiological biomarker study) |
| Tazy et al. | 2019 | Design and Testing of Electronic Nose for Determining the Pattern of Bad Breath Classification in Patients with Diabetes Mellitus and Pulmonary Tuberculosis (TBC) | Publication type |
| Chen et al. | 2020 | Detection of Tuberculosis by The Analysis of Exhaled Breath Particles with High-resolution Mass Spectrometry | No diagnostic performance analysis (proof-of-concept) |
| Patterson et al. | 2020 | Sensitivity optimisation of tuberculosis bioaerosol sampling | Study design (non-clinical experiment) |
| Bobak et al. | 2021 | Breath can discriminate tuberculosis from other lower respiratory illness in children | Study design (unconfirmed TB without reference standard) |
| Dinkele et al. | 2021 | Capture and visualization of live Mycobacterium tuberculosis bacilli from tuberculosis patient bioaerosols | Study design (capture and visualization of *M.* *tuberculosis* bioaerosols) |
| Saktiawati et al | 2021 | eNose-TB: A trial study protocol of electronic nose for tuberculosis screening in Indonesia | Publication type |
| Saravanakumar et al. | 2021 | Artificial intelligence for a bio-sensored detection of tuberculosis | Study design (non-clinical experiment) |
| Teixeira et al. | 2021 | The electronic nose as a rule-out test for tuberculosis in an indigenous population | Population not eligible (community screening) |
| Ma et al. | 2022 | Rapid detection of airborne protein from Mycobacterium tuberculosis using a biosensor detection system | No diagnostic performance analysis (laboratory prototype) |
| Fu et al. | 2023 | Detecting latent tuberculosis infection with a breath test using mass spectrometer: A pilot cross-sectional study | Population not eligible (latent TB) |
| Cheng et al. | 2024 | Visual detection of Mycobacterium tuberculosis in exhaled breath using a N95 enrichment respirator, RPA, and lateral flow assay | Study design (non-clinical experiment) |
| Happaerts et al. | 2024 | Exploring the use of exhaled breath as a diagnostic tool for pulmonary TB | Publication type |
| Nijman et al. | 2025 | Broadening the diagnostic landscape of *Mycobacterium tuberculosis* infection: analyzing exhaled breath | Publication type |

*Reasons for exclusion were classified according to PRISMA 2020:

**(1) Study design** – conceptual or laboratory prototype development, in silico modeling, simulation, or feasibility studies without clinical validation or patient-level data.

**(2) Population not eligible** – studies not involving individuals with suspected or confirmed pulmonary TB, or those not aligned with the PCCframework.

**(3) Publication type** – conference abstracts, narrative reviews, protocols, or other non–peer-reviewed formats lacking sufficientmethodological detail.

**(4) No diagnostic performance analysis** – studies that did not report clinical accuracy metrics (e.g., sensitivity, specificity, AUC, accuracy) for active pulmonary TB.

**Table S4.** Diagnostic accuracy of exhaled breath-based tests: sensitivity, specificity, and 95% confidence intervals (CIs) as reported by included studies

| Study | Sensitivity (%) | 95% CI Sensitivity | Specificity (%) | 95% CI Specificity |
| --- | --- | --- | --- | --- |
| Badola, 2023 [17] | 95.7 | 90.8-98.4 | 91.3 | 86.4-94.8 |
| Beccaria, 2019 [18] | 100 | Not reported^1^ | 60 | Not reported^1^ |
| Bruins, 2013 [19] | 76 | 72.6-80.1 | 87.2 | 85.8-88.6 |
| Chen, 2022 [20] | Not reported^1^ | Not reported^1^ | Not reported^1^ | Not reported^1^ |
| Fu, 2023 [21] | 91.7 | 87.4-96 | 93 | 90-96.1 |
| Kolk, 2012 [22] | 62 | 41.2-82.8 | 84 | 70-98 |
| Mohamed, 2017 [23] | 98.5 | 92.1-100 | 100 | 93.5-100 |
| Mosquera, 2022 [24] | 93.4 | Not reported^1^ | 100 | Not reported^1^ |
| Mougang, 2023 [25] | 90.8 | 73.8-100 | 85.7 | 67.7-100 |
| Phillips, 2010 [26] | 84 | Not reported^1^ | 64.7 | Not reported^1^ |
| Phillips, 2012 [27] | 71.2 | Not reported^1^ | 72 | Not reported^1^ |
| SaktiawatiI, 2019 [28] | 78 | 70-85 | 42 | 34-50 |
| Coronel Teixeira, 2017 [29] | 88 | Not reported^1^ | 92 | Not reported^1^ |
| Coronel Teixeira, 2023 [30] | 52.3 | 39.6-64.7 | 36.4 | 12.4-68.4 |
| Zetola, 2017 [31] | 94.1 | 83.8-98.8 | 90 | 68.3-98.8 |
| Bijker, 2024 [32] | 86 | 62-96 | 42 | 30-55 |
| Xu, 2024 [33] | 88.5 | 76.9-100 | 100 | 82.9-100 |
| Meiwes, 2024 [34] | 0 | 0-0 | Not reported^1^ | Not reported^1^ |
| Alfahdawi, 2025 [35] | 100 | Not reported^1^ | 100 | Not reported^1^ |

^1^ Confidence intervals (95%) were not reported by the original authors. The table presents only data explicitly reported by included studies. No additional calculations were performed.
